# Pharmacological interventions for lipid transport disorders

**DOI:** 10.3389/fnins.2023.1321250

**Published:** 2023-12-14

**Authors:** Aaron M. Neiman

**Affiliations:** Department of Biochemistry and Cell Biology, Stony Brook University, Stony Brook, NY, United States

**Keywords:** membrane contact site (MCS), *VPS13* genes, lipid transport, proteolysis-targeting chimeric (PROTAC) molecule, neuroacanthocytosis, Parkinson’s disease

## Abstract

The recent discovery that defects in inter-organelle lipid transport are at the heart of several neurological and neurodegenerative disorders raises the challenge of identifying therapeutic strategies to correct lipid transport defects. This perspective highlights two potential strategies suggested by the study of lipid transport in budding yeast. In the first approach, small molecules are proposed that enhance the lipid transfer activity of *VPS13* proteins and thereby compensate for reduced transport. In the second approach, molecules that act as inter-organelle tethers could be used to create artificial contact sites and bypass the loss of endogenous contacts.

## Introduction

**Figure 1:**
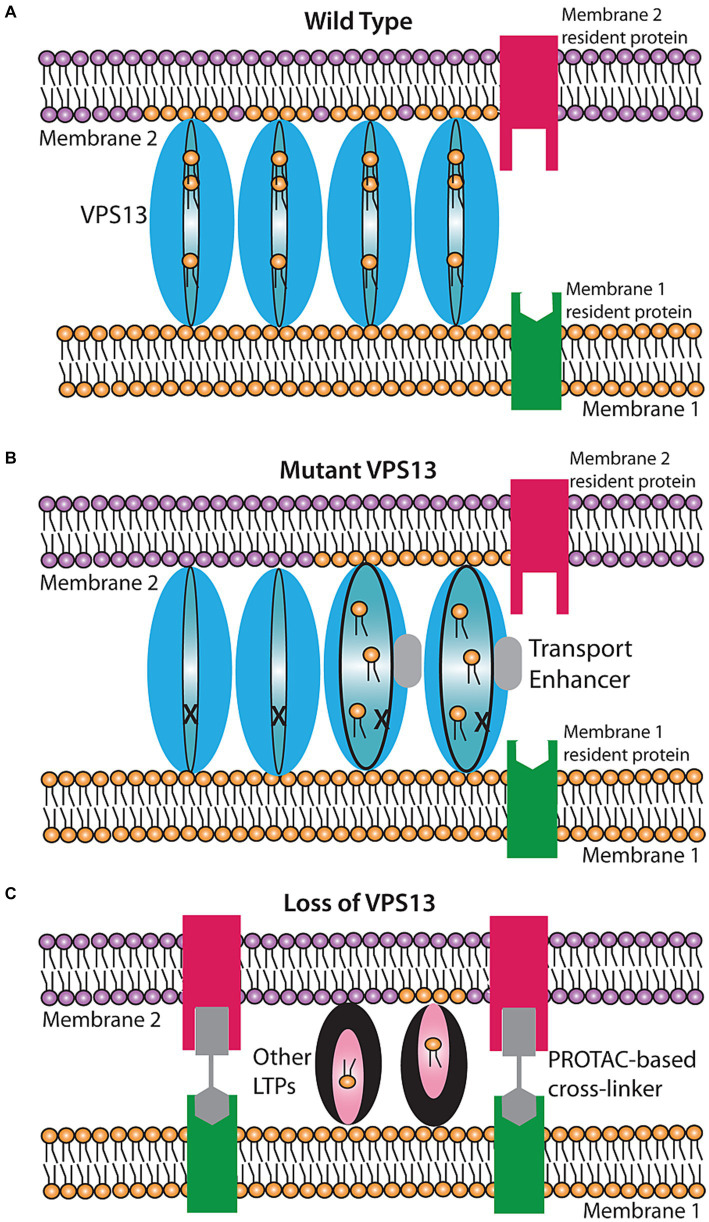
Proposed models for rescue of lipid transport in VPS13 mutant cells. **(A)** Wild-type transport at a VPS13-mediated contact site. VPS13 protein (blue oval) can transfer lipids between membranes through a hydrophobic channel in the protein. **(B)** Rescue by a transport enhancer. In the presence of a defective VPS13 (indicated by the ‘X’), transport is stopped. A transport enhancer (gray box) triggers a conformational change in the VPS13 protein restoring transport function. **(C)** Rescue by a bypass agent. In cells entirely lacking VPS13, new contact sites can be created by introducing a bifunctional cross linker that creates an artificial contact site between the two membranes. Other lipid transport proteins (LTPs) in the cell can then mediate lipid transport at these artificial contacts.

Mutations in the *VPS13* gene family in humans (*VPS13A*, *B*, *C* or *D*) are associated with VPS13A disease, Cohen Syndrome, Parkinson’s disease or cerebellar ataxia, respectively (Holmes et al., 2001; Rampoldi et al., 2001; Kolehmainen et al., 2003; Hayflick et al., 2013; Lesage et al., 2016; Gauthier et al., 2018; Seong et al., 2018). Recently it was discovered that *VPS13* genes encode proteins that bind the membranes of different organelles and mediate transfer of lipids between those membranes through a hydrophobic channel within the VPS13 protein (Figure 1A). These findings suggest that a failure in lipid transport is the basis of the different VPS13 family diseases and that the proper lipid composition of different organelles is critical to prevent these disorders.

The recognition of the molecular function of the VPS13 family raises an important general question in the potential treatment of this class of diseases – how can pharmacological intervention be used to reverse the cellular effects of a lipid transport defect? The purpose of this perspective is to consider potential strategies for the development of pharmaceuticals that can directly restore lipid transport and thereby address the root cause of the disease. I highlight results from studies using the yeast model system that suggest two possible avenues for the development of such drugs.

### Strategy 1: lipid transport enhancers: improving the function of mutant VPS13 proteins

In yeast there is a single *VPS13* gene that is required for multiple, independent processes, including spore formation, vacuolar sorting, mitochondrial integrity and endoplasmic reticulum (ER)-phagy (Bankaitis et al., 1986; Park and Neiman, 2012; Park et al., 2016; Chen et al., 2020). For example, the Vps13 protein acts in parallel with a different lipid transfer protein complex, termed ERMES, to mediate lipid transport between mitochondria and other organelles (Lang et al., 2015; Park et al., 2016). While mutants that individually abolish ERMES or Vps13 function are viable, loss of both ERMES and Vps13 function is lethal (Lang et al., 2015; Park et al., 2016). Several groups have identified mutations in the *VPS13* gene that compensate for the loss of ERMES, restoring normal growth (Lang et al., 2015; Park et al., 2016; van Leeuwen et al., 2016). These mutations create dominant, gain-of-function *VPS13* alleles, one of which is *VPS13-G718K*. One explanation for the ability of the dominant *VPS13* mutants to suppress the phenotypes associated with lack of ERMES is that the mutations increase Vps13-mediated delivery of lipids to mitochondria to obviate the need for ERMES.

The dominant *VPS13* mutations not only compensate for the loss of ERMES, they can act as intragenic suppressors of non-null *vps13* alleles as well (Park et al., 2016, 2021). For example, some patients with VPS13A disease carry a substitution of leucine 67 to proline in VPS13A. Mutating the corresponding leucine codon in yeast *VPS13* to proline creates the *vps13-L66P* allele that is synthetically lethal in combination with an ERMES mutant (Tomiyasu et al., 2011; Park et al., 2016). Introduction of a dominant gain-of-function *VPS13* mutation such as *G718K* into *vps13-L66P* restores growth in the absence of ERMES, perhaps by increasing levels of Vps13-mediated lipid transport. Similarly, when a cognate mutation to one in *VPS13D* identified in patients with cerebellar ataxia is introduced into yeast *VPS13* (*vps13-N2428S*), proper delivery of cargoes to the yeast vacuole is disrupted (Gauthier et al., 2018; Park et al., 2021). Introduction of the *G718K* mutation into the *vps13-N2428S* allele partially relieves this trafficking defect. Thus, in the context of the yeast cell, these mutations can promote the function of a mutant Vps13 protein at different contact sites, possibly by increasing the efficacy of lipid transfer.

An important point is that these suppressor mutations are relatively easy to obtain. Nineteen different suppressor mutations have been identified and they map throughout the N-terminal half of the Vps13 protein, particularly along the portion that forms the lipid transport channel (Lang et al., 2015; Park et al., 2016; van Leeuwen et al., 2016; Hanna et al., 2023). If these mutations promote a conformation that is permissive for lipid transfer, it may be possible to identify pharmacological agents that similarly enhance lipid transport by mimicking the effect of these mutations on Vps13 protein conformation (Figure 1B). Based on the yeast results, such agents would be expected to partially or fully compensate for the cellular effects of a non-null allele of *VPS13*.

Such transport-enhancing agents may be particularly useful for *VPS13D* disorders where the alleles found in patients thus far are primarily missense mutations (Gauthier et al., 2018; Seong et al., 2018). Knockouts of *VPS13D* in *Drosophila* or mice are embryonic lethal (Anding et al., 2018; Gauthier et al., 2018; Seong et al., 2018), which suggests that the human *vps13D* mutant alleles found in patients are likely partially functional and so a drug that increased their activity could be an effective treatment. For the other human *VPS13* family genes, most of the alleles isolated from patients are null mutations that eliminate the protein (Dobson-Stone et al., 2002; Seifert et al., 2009; Monfrini et al., 2022). Even in these instances, however, there is the possibility that activating one member of the VPS13 family might be able to partially compensate for the loss of another. This is particularly true for VPS13A and VPS13C, which are the most closely related pair evolutionarily and have some overlap in the contact sites they occupy (Kumar et al., 2018; Chen et al., 2022).

### Strategy 2: bypass agents: induction of alternative contact sites

For patients who carry null mutants of VPS13 family genes, what is needed is not an agent that increases the activity of a mutant protein but one that restores lipid transfer in the absence of the protein entirely. Results from yeast have shown that such a ‘bypass agent’ is obtainable. The role of ERMES is to create both a connection between ER and mitochondrial membranes and to shuttle lipids between those membranes at this contact site (Kornmann and Walter, 2010). Kornmann and colleagues created cells containing a synthetic protein with an N-terminal transmembrane domain anchored in the ER and a C-terminal transmembrane in the mitochondrial outer membrane (Kornmann et al., 2009). This construct holds the two membranes in close apposition creating artificial contact sites. Remarkably, even though this construct has no capacity to transfer lipids, its expression is sufficient to suppress the phenotypes of loss of ERMES (Kornmann et al., 2009). Thus, artificially anchoring the two membranes together must allow other lipid transfer activities in the cell to mediate the necessary movement of lipids. Whether such a bypass is unique to ERMES or can also occur for other (e.g., VPS13-mediated) contacts is not known, but well worth exploring. In principle, artificial tethers targeted to appropriate pairs of membranes could act as bypass agents to restore lipid transfer in the absence of VPS13 proteins (Figure 1C).

Protein-based interorganelle tethers have already been shown to work in human cells (Varnai and Balla, 1998; Chang et al., 2013; Jing et al., 2020). However, the use of protein-based tethers would be difficult therapeutically as the problems associated with delivering and expressing the proteins in the appropriate tissues would be significant. What is needed is a chemical agent that can be used to connect two membranes *in vivo*. Recently, therapeutics have been developed for use in a different context that could be employed to create artificial contact sites.

Proteolysis targeting chimeras (PROTACs) have been developed as a means of targeting specific proteins for ubiquitin mediated degradation in the cell (Neklesa et al., 2017; Liu et al., 2022). These molecules consist of three moieties; a ligand that binds specifically to an E3 ligase, a linker region whose length can be varied, and a second ligand that binds to the protein that is to be targeted for degradation. This system has been developed to eliminate disease-causing proteins, for example activated kinases or misfolded proteins, from the cell by linking the target to the ubiquitin ligase and thereby increasing its rate of turnover. This approach has shown great promise and several different PROTAC-based treatments are currently in clinical trials (Liu et al., 2022; Weng et al., 2023).

A variety of linkers and ligands have already been developed (Weng et al., 2023). Importantly, as the proteins to be degraded are often well-established drug targets, the ligand moieties take advantage of known drugs with high affinity to specific molecules making the pharmacological efficacy of the PROTAC more likely (Weng et al., 2023). To adapt this system to contact sites is simply a matter of placing ligands that bind to two different integral membrane proteins of different organelles at the ends of the linker. For example placing ligands for a plasma membrane protein and an ER protein at either end of the linker would create a molecule that when added to cells should induce ER-Plasma membrane junctions.

### Technical challenges for drug development

The dominant mutations identified in yeast are often substitutions of small residues such as glycine for large, charged residues such as arginine or glutamate (Lang et al., 2015; Park et al., 2016). Using structural models for the Vps13 proteins, it should be possible to design candidate molecules that bind and mimic the effects of introducing charges at these positions in the molecule (Batool et al., 2019). Even with a rational drug design approach, however, it will be necessary to screen through many candidate compounds. Thus, an important challenge will be to create cell-based assays for VPS13 protein function that allow for rapid screening of candidates. Currently, such assays exist for yeast, but appropriate cell lines and assays still need to be developed for the different human VPS13 family members.

For bypass agents, the use of the PROTAC platform would greatly speed the design of candidate molecules. For instance, ligands binding the plasma membrane protein CCR9 and the ER protein HMG-CoA reductase have already been incorporated into PROTACs (Li et al., 2020; Huber et al., 2022). Synthesis of a molecule with these ligands at either end should allow a rapid test of the ability of this approach to generate novel ER plasma membrane contacts. It should be noted, however, that these ligands might interfere with the function CCR9 and HMG-CoA reductase, respectively, creating unwanted effects. It will be important to ensure that the ligands used do not create cellular phenotypes on their own. Moreover, for protein-based interorganelle tethers it has been found that the length of the tether can have significant effects on its functionality (Varnai et al., 2007). Thus, it will be necessary not only to find appropriate ligands for each end of the PROTAC molecule but also to empirically test different lengths of linker. Again, the development of rapid phenotypic assays in knockout cell lines will be a critical tool for this process.

## Discussion

Finally, to implement either of these strategies to address a specific disorder, more understanding of the cellular role of each VPS13 family protein is still required. For instance, in order to design PROTACs to connect the appropriate pair(s) of organelles, we will first need to know loss of which membrane contacts are relevant in each VPS13 family disease. However, this information is not necessary to begin developing and testing strategies to stimulate lipid transfer between any given pair of membranes. This will speed the development of treatments even as our understanding of the basis of these diseases continues to grow.

## Data availability statement

The original contributions presented in the study are included in the article/supplementary material, further inquiries can be directed to the corresponding author.

## Author contributions

AN: Writing – original draft, Writing – review & editing.
